# A retrospective study of the impact of health worker strikes on maternal and child health care utilization in western Kenya

**DOI:** 10.1186/s12913-021-06939-7

**Published:** 2021-09-01

**Authors:** Michael L. Scanlon, Lauren Y. Maldonado, Justus E. Ikemeri, Anjellah Jumah, Getrude Anusu, Jeffrey N. Bone, Sheilah Chelagat, Joann Chebet Keter, Laura Ruhl, Julia Songok, Astrid Christoffersen-Deb

**Affiliations:** 1 Indiana University Center for Global Health, 702 Rotary Circle, Suite RO 101, Indianapolis, Indiana USA; 2 Academic Model Providing Access to Healthcare (AMPATH), Eldoret, Kenya; 3 Department of Medicine and Pediatrics, Massachusetts General Hospital, Boston, MA USA; 4 Department of Obstetrics and Gynaecology, University of British Columbia, Vancouver, Canada; 5 Department of Medicine, Indiana University School of Medicine, Indianapolis, IN USA; 6 Department of Child Health and Paediatrics, College of Health Sciences, School of Medicine, Moi University, Eldoret, Kenya; 7 Department of Obstetrics and Gynaecology, University of Toronto, Toronto, Canada

**Keywords:** Health worker strike, Maternal child health, Kenya

## Abstract

**Background:**

There have been dozens of strikes by health workers in Kenya in the past decade, but there are few studies of their impact on maternal and child health services and outcomes. We conducted a retrospective survey study to assess the impact of nationwide strikes by health workers in 2017 on utilization of maternal and child health services in western Kenya.

**Methods:**

We utilized a parent study to enroll women who were pregnant in 2017 when there were prolonged strikes by health workers (“strike group”) and women who were pregnant in 2018 when there were no major strikes (“control group”). Trained research assistants administered a close-ended survey to retrospectively collect demographic and pregnancy-related health utilization and outcomes data. Data were collected between March and July 2019. The primary outcomes of interest were antenatal care (ANC) visits, delivery location, and early child immunizations. Generalized estimating equations were used to estimate risk ratios between the strike and control groups, adjusting for socioeconomic status, health insurance status, and clustering. Adjusted risk ratios (ARR) were calculated with 95% confidence intervals (95%CI).

**Results:**

Of 1341 women recruited in the parent study in 2017 (strike group), we re-consented 843 women (63%) to participate. Of 924 women recruited in the control arm of the parent study in 2018 (control group), we re-consented 728 women (79%). Women in the strike group were 17% less likely to attend at least four ANC visits during their pregnancy (ARR 0.83, 95%CI 0.74, 0.94) and 16% less likely to deliver in a health facility (ARR 0.84, 95%CI 0.76, 0.92) compared to women in the control group. Whether a child received their first oral polio vaccine did not differ significantly between groups, but children of women in the strike group received their vaccine significantly longer after birth (13 days versus 7 days, *p* = 0.002).

**Conclusion:**

We found that women who were pregnant during nationwide strikes by health workers in 2017 were less likely to receive WHO-recommended maternal child health services. Strategies to maintain these services during strikes are urgently needed.

## Background

Strikes by health workers are not new phenomena but there is evidence that they may be increasing globally and represent an important but under-explored challenge to initiatives for universal health coverage [1]. In the past decade, health workers in Kenya have launched major strikes in the public health sector to protest a wide range of issues, including low pay, poor working conditions, and implementation of collective bargaining agreements, among others [2]. In 2017, public sector physicians, nurses, and clinical officers were on separate nationwide strikes for a combined 250 days. Physicians were on strike for 100 days (December 5, 2016 to March 15, 2017) and nurses were on strike for 150 days (June 5, 2017 to November 2, 2017). Clinical officers, who are mid-level, non-physician clinicians in Kenya [3], launched their own 20-day strike in the midst of the nurses’ strikes. Strikes in Kenya are associated with significant decreases in inpatient and outpatient admissions in public facilities [4, 5], but their association with health outcomes including morbidity and mortality is less clear [6–8]. The impact of strikes on essential maternal and child health services and outcomes has not been adequately explored [9].

Maternal and neonatal mortality is a major public health issue in Kenya [10, 11]. In 2017, the maternal mortality ratio was estimated to be 342 per 100,000 live births [12], and data from the most recent Demographic and Health Survey in 2014 found that only about 60% of women attended four or more antenatal care (ANC) visits and delivered with a skilled birth attendant [13]. Recent government reforms have aimed to improve access and utilization of maternity services [14]. In 2017, the government launched a new scheme called Linda Mama as part of its strategy to progress towards universal health coverage. The program is eligible to all Kenyan citizens and covers maternity services including ANC, delivery, and postnatal care under the national health insurer, the National Hospital Insurance Fund, at accredited public and private health facilities [15]. Data show that these policies have likely contributed to increases in ANC and delivery with a skilled birth attendant, but implementation of these policies has been inconsistent due to significant demand- and supply-side barriers, including systemic inequities particularly for women who are poorer and reside in more rural communities [16–20]. It is unclear how recent strikes have affected the implementation of these policies and potentially exacerbated inequities in service coverage and utilization.

To investigate the impact of the prolonged strikes by health workers in Kenya on maternal and child health care utilization, we conducted a natural experiment using retrospective surveys with women who were pregnant in 2017 during health worker strikes and women who were pregnant in 2018 when there were no major strikes.

## Methods

### Study setting

This study was conducted in Trans Nzoia County in western Kenya. Trans Nzoia has a population of approximately one million people with a largely rural and agricultural economy, and generally scores below national averages on primary and maternal child health indicators such as ANC, facility deliveries, and child immunizations [21]. In the most recent Kenya Demographic and Health Survey, the percentage of women who delivered in a health facility was 41.5% in Trans Nzoia compared to 61.2% nationally [13]. According to the Kenya Ministry of Health Master Health Facility List, Trans Nzoia has 182 registered health facilities, which includes seven public hospitals, five private hospitals, one faith-based hospital, and one County referral hospital [22]. In addition to national-level strikes by health workers, Trans Nzoia nurses employed in public sector facilities launched their own 44-day strike across the County from February 1, 2017 to March 23, 2017 to protest delays in salaries, remittance of statutory deductions, and promotions [23]. This meant that in Trans Nzoia either physicians, nurses, or clinical officers were on strike in the public health sector for a total of 232 days in 2017. We are not aware of any major strikes by health workers in private or faith-based facilities during the study period.

### Study design

We utilized a parent study to conduct a natural experiment and retrospectively assessed the impact of the 2017 strikes on the utilization of maternal and child health services. As part of the parent study called *Chamas for Change*, we recruited a cohort of pregnant women in 2017 and a different cohort of pregnant women in 2018 when there were no major strikes by health workers [24]. In 2019, we traced women previously recruited in the parent study, re-consented them in the current study, and administered a close-ended questionnaire. We also conducted focus group discussions with a subset of these women and community health volunteers (CHVs) as well as in-depth interviews with health facility managers, which is reported elsewhere [25].

The parent study, *Chamas for Change*, was a cluster randomized controlled trial to evaluate the effectiveness of a CHV-led, group-based model of care to improve maternal and child health in Trans Nzoia County in western Kenya. In 2017, pregnant women who presented for ANC services at one of 74 public or private health facilities in Trans Nzoia were recruited into the parent study. All participants provided informed consent to participate in the parent study and completed a baseline assessment. Prior to launching the intervention, nurses launched their nationwide strike, and the parent study was halted. In 2018, a new cohort of pregnant women was recruited to participate in the trial using the same enrollment criteria. Additional details on the parent study are provided elsewhere [24].

### Participants

Participants were women who were recruited in the *Chamas for Change* parent study in 2017 (“strike group”) and in the control arm of the trial in 2018 (“control group”). Women in the strike group and the control group did not receive any intervention as part of the parent study and met the same inclusion criteria at the time of enrollment into the parent study: 18 years of age or older, pregnant, and less than 24 weeks gestation based on last menstrual period. Using contact information provided as part of the parent study, we contacted women to see if they would be interested in participating in this study. Participants completed an interview using a close-ended questionnaire about their pregnancy and pregnancy-related health utilization and outcomes with a trained research assistant.

### Data collection

Data were collected between March and July 2019. All participants provided informed consent to participate. Interviews using close-ended questionnaires were conducted in English or Kiswahili by trained research assistants at participants’ homes or by phone if they had moved outside of Trans Nzoia. All data were collected via computer tablets using a mobile REDCap survey application and synchronized to a central REDCap database twice weekly [26, 27]. The interview took approximately 30 min to complete. Demographic and socioeconomic characteristics were collected in addition to data on pregnancy-related services and outcomes. For in-person interviews, women were asked to share their Mother-Baby Booklet that is provided by the Kenya Ministry of Health that contains pregnancy and delivery information. In cases where the Mother-Baby Booklet was not available and for participants who were contacted by phone, women were asked to self-report.

### Analysis

Data were analyzed descriptively for demographic, socio-economic, and pregnancy-related outcomes. We compared demographics and socio-economic status between women in the strike versus control group using Student’s t-tests for normally distributed continuous variables and Pearson’s chi-squared tests for categorical variables. Generalized estimating equations (to account for clustering) with a Poisson link were used to estimate the risk ratios between the strike and control group for outcomes of interest [28]. As in the parent study [24], the primary outcomes of interest were ANC visits, facility-based delivery, and early child immunizations. There were high rates of missing immunization data (> 50%) other than the first oral polio vaccine, and so additional immunizations were excluded in the analysis. Models were adjusted for socioeconomic status and insurance status at delivery. For continuous outcomes, a Gaussian link rather than a Poisson was used. Adjusted risk ratios (ARR) are presented with 95% confidence intervals (95%CI).

## Results

### Participant characteristics

Of 1341 women recruited in the parent study in 2017 (strike group), we successfully located and re-consented 843 women (63%) to participate in close-ended interviews using the contact information provided during their initial enrollment. Of 924 women recruited in the control arm of the parent study in 2018 (control group), 728 women (79%) were located and re-consented. The average number of days between delivery and the study interview was 611 days (standard deviation, SD, 63 days) for women in the strike group and 324 days (SD 68 days) for women in the control group. Most interviews were conducted in-person by research assistants (84% in the strike group versus 88% in the control group) with the remainder conducted by phone.

Demographic characteristics of participants who completed close-ended interviews are presented in Table 1. There were no meaningful differences in age, marital status, employment status, or poverty level between women in the strike versus control group. Women in the control group were significantly more likely to have health insurance at the time of delivery compared to women in the strike group (59% versus 15%). The majority of women in the strike group (76%) reported being aware of a health worker strike during their pregnancy. In the control group, 4% of women also reported being aware of a health worker strike during their pregnancy even though we could not find any report of strikes in this period. Most women (78%) in the strike group gave birth during the nurses’ strike from June 5 to November 2, 2017, with the remainder either having a missing delivery date or reporting a delivery date after the nurses’ strike ended.

**Table 1:** Characteristics of participants who completed close-ended interview

Characteristic	Strike group (women pregnant in 2017) *n* = 843	Control group (women pregnant in 2018) *n* = 728	*p*-value
% reconsented from parent study	63%	79%	n/a
Mean age, years (SD)	27.0 (5.9)	27.1 (6.2)	0.83
Marital status, married	684 (81%)	606 (83%)	0.74
Employed (formally or informally)	349 (41%)	272 (37%)	0.10
Mean poverty index score (SD) ^a^	50.6 (20.0)	48.8 (20.1)	0.07
Health insurance at delivery	120 (15%)	412 (59%)	< 0.01

^a^ We used the 10-item Poverty Probability Index for Kenya (https://www.povertyindex.org/country/kenya) created by Innovations for Poverty Action (version October 2018) that is based on Kenya’s 2015 Integrated Household Budget Survey

### Maternal and child health care utilization and outcomes

Women in the strike group were 17% less likely to attend at least four ANC visits during their pregnancy (ARR 0.83, 95%CI 0.74, 0.94) and 16% less likely to deliver in a health facility (ARR 0.84, 95%CI 0.76, 0.92) compared to women in the control group (Table 2). Among women who delivered in a health facility, women in the strike group were more likely to deliver in a private facility (38%) compared to women in the control group (11%). Only 33% of women in the strike group reported that they delivered their child at their preferred location compared to 73% of women in the control group. There were no differences in livebirth or first oral polio vaccine (OPV 0) rates. Data for complications during delivery and CHV visit within 48 hours of birth were inconclusive. Children of women in the strike group received their first OPV 0 vaccine significantly later after birth compared to the control group (13 days versus seven days, mean difference = 8.02, 95% CI = 3.06, 12.98).

**Table 2:** Maternal and child health outcomes

Outcome	Strike group (women pregnant in 2017) *n* = 843	Control group (women pregnant in 2018) *n* = 728	Adjusted risk ratio [95% CI]; *p*-value ^d^
4 or more ANC visits, n (%)	463 (55%)	507 (70%)	**0.83 [0.74,0.94]; 0.004**
Delivered in a facility (public or private), n (%)	452 (55%)	514 (73%)	**0.84 [0.76,0.92]; < 0.001**
Delivery location, n (%)			n/a
Home alone	75 (9%)	43 (6%)	
Home with TBA	284 (34%)	139 (20%)	
In transit	14 (2%)	8 (1%)	
Private facility	316 (38%)	77 (11%)	
Public facility	136 (17%)	437 (62%)	
Delivered at preferred location, n (%)	268 (33%)	514 (73%)	**0.49 [0.42,0.58]; < 0.001**
Any delivery complications, n (%) ^a, b^	113 (19%)	132 (25%)	0.81 [0.59,1.11]; 0.185
Delivered a live child, n (%)	794 (94%)	696 (96%)	1.00 [0.97, 1.02]; 0.711
Mean child birthweight, kgs (SD)	3.40 (0.66)	3.43 (0.76)	−0.04 [−0.13,0.06]; 0.442 ^c^
CHV visited home within 48 hours of birth, n (%)	105 (13%)	97 (14%)	0.98 [0.68,1.43]; 0.92
Child received OPV 0 vaccine, n (%) ^b^	474 (83%)	468 (89%)	0.98 [0.93,1.03]; 0.356
Median days from birth to child receiving OPV 0 vaccine ^b^	13	7	**8.02 [3.06,12.98]; 0.002**^**c**^
Child alive at time of interview, n (%)	772 (98%)	675 (98%)	1.00 [0.98,1.02]; 0.942

^a^ Delivery complications included “bled a lot,” “baby got stuck,” “infection,” “high blood pressure,” “seizures,” “baby needed resuscitation/help to breath,” or other complication specified by the participant

^b^ Significant missing data (between 29 and 44% missing). For all other variables, missingness was < 5%

^c^ Mean difference with 95%CI was used rather than risk ratios

^d^ Risk ratios were adjusted by employment status (employed vs. unemployed), mean poverty index score, and insurance status (insured vs. uninsured) at the time of delivery

## Discussion

We found that recent strikes by health workers were associated with lower maternal and health care utilization of ANC and delivery at a health facility among pregnant women in Trans Nzoia County in western Kenya. We also found evidence that strikes were associated with delayed child immunization for their first oral polio vaccine. Several recent studies have explored the impact of strikes by health workers on health services and outcomes in Kenya [4–9]. These studies mostly rely on hospital and inpatient data, and our study contributes important community-level data on outpatient and antenatal care to the literature. A study using hospital admissions data at 13 County referral hospitals during the 2016–2017 nationwide physicians’ and nurses’ strikes found that inpatient admissions decreased significantly during strike months, with the authors estimating that 183,170 individuals were expected but did not receive inpatient care at these hospitals over the course of the strikes, including 60,000 maternity patients [4]. Another study using records from 18 County referral hospitals and 14 faith-based health facilities found that the proportion of fully immunized infants fell 57% in public facilities and increased 252% in faith-based ones during the 2017 nurses’ strike [9]. In our study, a lower proportion of children received their first oral polio vaccine in the strike group (83% versus 89%) but this was not statistically significant, although children in the strike group did receive their vaccine significantly later after birth.

While our study was not designed to detect differences in maternal or child mortality, studies reported mixed findings on the association between strikes by health workers and mortality in Kenya [6, 7]. In the only study to date to use population-level health outcomes data, during six strikes by health workers ranging from 9 to 42 days between 2010 and 2016 in Kilifi County, Kenya there was no change in all-cause or cause-specific mortality during strike versus non-strike days [8]. The authors suggest a number of potential explanations for their findings including service delivery continuing in private and faith-based facilities, relatively short duration of strikes, decreased exposure to poor quality inpatient care and high-risk procedures, and the fact that a large proportion of deaths (during strike and non-strike periods) occur outside of health facilities. Population-level data on maternal and child health utilization and outcomes during the study period were not publicly available at the time of writing, but these data will provide important additional insights into the impact of strikes in 2017.

Given that the strikes in 2017 lasted longer than previous strikes in Kenya, it is possible that they were more likely to affect population-level mortality that is not captured by studies using inpatient data [29, 30]. Decreased ANC coverage during strikes could have also interrupted prevention of mother-to-child transmission services for women living with HIV, but there are no published studies investigating strike-related changes in mother-to-child transmission of HIV. However, even in the control group, our study found that only 70% of participants attend 4 or more ANC visits and only 73% delivered at a health facility with a skilled health worker when there were no health worker strikes. Several studies have suggested that the poor quality of facility-based maternal and child health care may account for slower reductions in maternal and neonatal mortality compared to increases in service utilization and facility-based care [11, 31–33]. Research also shows that perceptions of poor quality maternal and child health services among mothers, including long wait times, lack of providers and essential equipment and drugs, disrespectful care, and out-of-pocket payments, represent important barriers to seeking care [34].

It is important to note that many of issues related to poor quality of care and ill-equipped providers and facilities have been at the center of labor disputes and recent strikes in Kenya, with health workers’ unions demanding increased investment in the public health sector. It is unclear whether recent strikes have led to improvements in health care services for patients and improved working conditions for providers but addressing the underlying issues of strikes will be critical to efforts for health systems strengthening and universal health coverage, including maternal and child health care. Since this study, subsequent strikes by frontline health workers in Kenya as well as in countries around the world during the COVID-19 pandemic provide further evidence that strikes represent an important but understudied phenomenon in health systems and services research [35].

There are several limitations to this study. First, we relied on retrospective data from Mother-Baby Booklets and self-report to measure maternal and child utilization of services and outcomes. Significant missing data on early child immunizations meant that only data on the oral polio vaccine was included in the analysis, and we could not examine the impact of strikes on other immunizations across the first years of life. In addition to missing data, there was also the potential for recall bias for women who self-reported outcomes. We did not find evidence of increased pregnancy complications or child mortality, however, 37% of women from the parent study strike group were not able to be re-consented. The only demographic data available on missing women was age, which did not differ between missing and re-enrolled women. We were also not able to measure maternal mortality and it is possible that some women who were not able to be relocated had in fact died or had other complications.

Second, we present data from only one County in Kenya. While our findings are generally consistent with the literature available from other parts of Kenya, it is possible that maternal and child health services and outcomes were affected differently by strikes in other parts of Kenya. Trans Nzoia is poorer, more rural, and has generally worse maternal and child health indicators by comparison to Kenya overall, however, it does have a mostly representative split between public and private health facilities [22]. Third, we did not assess the impact of a specific health worker strike. Most participants in the strike group delivered during a nationwide nurses’ strike but were also pregnant at some point during the physicians’ strike earlier in 2017. Moreover, it is common in health facilities when one cadre of health worker is on strike, services may be suspended, and other cadres of health workers may not be working. Thus, it is difficult to assess exactly what health facilities and services remained functional at different times in 2017.

Finally, there were changes in the health system outside of the strike that may have affected maternal and child health services and utilization from 2017 to 2018 and utilization rates of health services tend to increase over time [36]. Thus, it is possible that some of the changes we observed were the result of temporal trends and policy changes. There are several reasons that we believe that temporal trends and policy changes do not wholly account for the significant differences in ANC and health facility deliveries we report between our strike and control study cohorts. As noted previously, in our study population only 15% of women had health insurance at the time of delivery in 2017, but 59% had health insurance at the time of delivery in 2018. This dramatic increase was likely due to the roll out of the Linda Mama insurance program that allows women to access maternity services for free in accredited public and private sector facilities, which was launched in 2017 but due to strikes and other implementation challenges most women were not able to enroll into the program until the following year [15]. To account for the potential impact of Linda Mama insurance enrollment on outcomes of interest, we adjusted for enrollment in health insurance as well as socioeconomic status in our analyses. Moreover, it is not clear that enrollment in Linda Mama has necessarily increased access to and utilization of services. A recent interrupted time-series analysis of ANC and facility deliveries in Kenya found that, controlling for periods of strikes by health workers, there were no significant changes in ANC or facility deliveries in public or private facilities between 2017 (when Linda Mama was launched) up to May 2019 [37]. Taken together with a study using two-year data pooled from 13 County public hospitals between January 2016 and December 2017 that shows a sharp decline and then rebound in admissions to maternity wards [4], our study provides further support that recent health workers’ strikes are associated with lower utilization of maternal and child health services.

## Conclusion

We found that access to and utilization of basic maternal and child health services were negatively impacted for pregnant women in Trans Nzoia during nationwide strikes by health workers in 2017. Our study findings are consistent with studies using inpatient and outpatient admissions data in Kenya and provide important community-level data on maternal and child health utilization. Strategies to maintain WHO-recommended maternal and child health services during strikes, particularly for those communities that rely on public sector services, are urgently needed.

## Data Availability

The dataset generated during the current study are not publicly available. The study dataset is available from the corresponding author on reasonable request.
